# Prevention and Treatment of Glucocorticoid-Induced Osteoporosis in Adults: Consensus Recommendations From the Belgian Bone Club

**DOI:** 10.3389/fendo.2022.908727

**Published:** 2022-06-09

**Authors:** Michaël R. Laurent, Stefan Goemaere, Charlotte Verroken, Pierre Bergmann, Jean-Jacques Body, Olivier Bruyère, Etienne Cavalier, Serge Rozenberg, Bruno Lapauw, Evelien Gielen

**Affiliations:** ^1^ Centre for Metabolic Bone Diseases, Department of Geriatrics, University Hospitals Leuven, Leuven, Belgium; ^2^ Department of Geriatrics, Imelda Hospital, Bonheiden, Belgium; ^3^ Unit for Osteoporosis and Metabolic Bone Diseases, Ghent University Hospital, Ghent, Belgium; ^4^ Department of Endocrinology and Metabolism, Ghent University Hospital, Ghent, Belgium; ^5^ Department of Nuclear Medicine, CHU Brugmann, Université Libre de Bruxelles, Brussels, Belgium; ^6^ Department of Medicine, CHU Brugmann, Université Libre de Bruxelles, Brussels, Belgium; ^7^ WHO Collaborating Center for Public Health Aspects of Musculoskeletal Health and Ageing, Division of Public Health, Epidemiology and Health Economics, University of Liège, Liège, Belgium; ^8^ Department of Clinical Chemistry, University of Liège, CHU de Liège, Liège, Belgium; ^9^ Department of Gynaecology and Obstetrics, Université Libre de Bruxelles, Brussels, Belgium; ^10^ Gerontology and Geriatrics section, Department of Public Health and Primary Care, University Hospitals Leuven and KU Leuven, Leuven, Belgium

**Keywords:** adults, Cushing syndrome, glucocorticoid-induced osteoporosis, glucocorticoids, osteoporosis, prevention

## Abstract

Glucocorticoids are effective immunomodulatory drugs used for many inflammatory disorders as well as in transplant recipients. However, both iatrogenic and endogenous glucocorticoid excess are also associated with several side effects including an increased risk of osteoporosis and fractures. Glucocorticoid-induced osteoporosis (GIOP) is a common secondary cause of osteoporosis in adults. Despite availability of clear evidence and international guidelines for the prevention of GIOP, a large treatment gap remains. In this narrative review, the Belgian Bone Club (BBC) updates its 2006 consensus recommendations for the prevention and treatment of GIOP in adults. The pathophysiology of GIOP is multifactorial. The BBC strongly advises non-pharmacological measures including physical exercise, smoking cessation and avoidance of alcohol abuse in all adults at risk for osteoporosis. Glucocorticoids are associated with impaired intestinal calcium absorption; the BBC therefore strongly recommend sufficient calcium intake and avoidance of vitamin D deficiency. We recommend assessment of fracture risk, taking age, sex, menopausal status, prior fractures, glucocorticoid dose, other clinical risk factors and bone mineral density into account. Placebo-controlled randomized controlled trials have demonstrated the efficacy of alendronate, risedronate, zoledronate, denosumab and teriparatide in GIOP. We suggest monitoring by dual-energy X-ray absorptiometry (DXA) and vertebral fracture identification one year after glucocorticoid initiation. The trabecular bone score might be considered during DXA monitoring. Extended femur scans might be considered at the time of DXA imaging in glucocorticoid users on long-term (≥ 3 years) antiresorptive therapy. Bone turnover markers may be considered for monitoring treatment with anti-resorptive or osteoanabolic drugs in GIOP. Although the pathophysiology of solid organ and hematopoietic stem cell transplantation-induced osteoporosis extends beyond GIOP alone, the BBC recommends similar evaluation, prevention, treatment and follow-up principles in these patients. Efforts to close the treatment gap in GIOP and implement available effective fracture prevention strategies into clinical practice in primary, secondary and tertiary care are urgently needed.

## 1 Introduction

Glucocorticoids are a group of endogenous hormones and/or drugs, which act mostly through the glucocorticoid receptor to exert anti-inflammatory, immunosuppressive, anti-proliferative and vasoconstrictive effects. Glucocorticoids are widely used for the treatment of acute and chronic inflammatory conditions, for the treatment of lymphoproliferative diseases, to alleviate side effects of chemotherapy or radiotherapy and to prevent rejection of organ transplants. Glucocorticoids are also used chronically as replacement therapy in patients with adrenal insufficiency.

About 1% of the adult population uses glucocorticoids chronically, particularly older adults (1). In large observational studies, ~3 % of post-menopausal women and older men were currently taking oral glucocorticoids (2, 3).

Chronic glucocorticoid excess increases the risk of adverse effects including both early and progressive bone loss, resulting in osteoporosis and increased risk of fractures. This also applies to endogenous or iatrogenic hypercortisolism in patients with Cushing’s syndrome, in whom side effects may be reverted upon curative treatment or dose reduction, respectively (4).

Glucocorticoid-induced osteoporosis (GIOP) is the most common drug-induced cause of secondary osteoporosis. In patients starting glucocorticoids, there is a rapid phase of bone loss (6-12% decline in lumbar spine bone mineral density [BMD] in the first year), followed by a slower but continuous decline thereafter (5, 6). This bone loss is most pronounced in regions of the skeleton with abundant trabecular bone, such as the lumbar spine (5, 7). Although vertebral fractures are particularly characteristic of GIOP (five-fold higher risk in high-dose glucocorticoid users), the risk of non-vertebral and hip fractures is also increased by ~65% and ~130%, respectively (8, 9). Glucocorticoid initiators have an annual incidence of ~5% for vertebral fractures and ~2.5% for non-vertebral fractures, while the risk of both types of fractures is about ~3% annually in chronic users (6). These findings suggest that glucocorticoid initiators are at imminent vertebral fracture risk (6). The economic burden of GIOP remains poorly characterized though it is likely substantial (10, 11).

Fracture risk increases early after initiation of glucocorticoid therapy (within the first three to six months), usually even before a substantial loss of BMD has become apparent (12). When glucocorticoids are discontinued, BMD may return towards baseline levels, while fracture risk decreases after ~1 year (5, 9, 12). Following glucocorticoid discontinuation BMD may only recover partially, depending on *e.g.* the underlying disease or the menopausal status in women. Fractures in GIOP occur at higher BMD than in postmenopausal osteoporosis (13). While some studies suggest that (current) daily glucocorticoid dose is the main determinant of fracture risk (14) others suggest that high cumulative doses (>1 g prednisolone equivalent) are an independent risk factor (9, 15). In long-term glucocorticoid users, the prevalence of fractures amounts to 30-50% (13). Also in endogenous Cushing syndrome, duration and severity of hypercortisolism has been associated with osteoporosis severity and fracture risk (16, 17).

The pathophysiology of GIOP is complex and consists of both direct effects on bone cells (osteoblasts, osteoclasts and osteocytes) and indirect effects through suppression of the somatotropic and gonadotropic axes, reduced intestinal calcium absorption and glucocorticoid-induced myopathy and cataracts, which increase the risk of falls (13). Glucocorticoids promote osteoclastogenesis and the survival of osteoclasts, stimulate adipogenesis, inhibit osteoblastogenesis and induce apoptosis of osteoblasts and osteocytes (13) in part *via* suppression of Wnt agonists (18) and upregulation of Wnt signaling inhibitors such as sclerostin and Dkk1 (19). Not only does this lead to reduced bone mass, they may also affect the unmineralized bone matrix, which may partly explain their BMD-independent effects (20, 21). Recent preclinical studies additionally show that glucocorticoid excess also disrupts the intestinal microbiota (18) endogenous biological rhythm of glucocorticoids, which may contribute to the detrimental effects on bone (22).

Glucocorticoids are prescribed for inflammatory or other conditions which themselves predispose to osteoporosis (23, 24). Therefore, fracture risk in glucocorticoid users may be drug-induced, related to the underlying disease, or both. In rheumatoid arthritis (RA) for example, patients requiring glucocorticoids have slightly lower spine and hip BMD than non-glucocorticoid users. About one-third of glucocorticoid-treated RA patients have osteoporotic BMD T-scores, and their prevalence of vertebral fractures is 6-20% (7). Still, in patients with early and active RA, meta-analyses of randomized controlled trials (RCTs) show no significant difference in BMD between glucocorticoid and placebo users through 24 months, suggesting that the positive effects on inflammation may counterbalance the direct adverse skeletal effects of glucocorticoids (25, 26). Cumulative glucocorticoid exposure also contributes to osteoporosis in systemic lupus erythematosus (27, 28), and psoriatic arthritis (29). Inflammatory bowel disease (IBD) itself is associated with lower BMD (30) but glucocorticoid use in IBD patients has been associated with an increased risk of vertebral fractures (31) as well as hip fracture in older IBD patients (32). Meta-analyses show that bisphosphonates prevent bone loss and vertebral fractures in patients with rheumatic diseases (33) (34) and IBD (35) but evidence in other chronic inflammatory conditions remains more limited.

Dosage of systemic exposure and duration of glucocorticoid treatment, but also age, low body mass index (BMI), underlying disease and low BMD are risk factors for glucocorticoid-induced fractures. Traditional clinical risk factors, such as previous fractures, family history of fractures, smoking, alcohol consumption and frequent falls independently contribute to fracture risk in persons taking glucocorticoids (36). The longer and the higher the glucocorticoid dose, the higher the risk of fractures. However, even long-term glucocorticoid doses < 5 mg prednisolone equivalent/day are associated with bone loss and fractures (8, 9). Repeated glucocorticoid courses also result in considerable cumulative exposure in older adults with asthma and chronic obstructive pulmonary disease (COPD), which contributes to the increased risk of osteoporosis and vertebral fractures in these conditions (9) (37–40).

Glucocorticoid exposure in children might impair peak bone mass and increase the risk of fractures later in life (41) although pediatric GIOP is outside the scope of this review. Osteoporosis in children and younger adults has been reviewed elsewhere (42).

The aim of this work is to provide up-to-date evidence-based recommendations for the prevention and treatment of GIOP in adults, building on our previous guidelines (43, 44). Since our previous recommendation from 2006, many relevant studies have been published and new treatments have become available (zoledronate, teriparatide, denosumab), while other recommendations covered drugs that are no longer recommended or available in Belgium (*e.g.* calcitonin, etidronate). The target audience are health professionals caring for adults at risk of GIOP, particularly in Belgium (although our work may be of interest for practitioners in other countries too). Of note, whereas some regulators distinguish between prevention of GIOP and treatment of (established) GIOP, we will not maintain this distinction and use both terms interchangeably, because we believe this distinction may be somewhat arbitrary and is not useful in clinical practice.

## 2 Materials and Methods

In order to obtain the best available evidence, the authors searched PubMed from 2005 (when our previous consensus recommendations were written (43) to March 31^st^, 2022 for existing systematic reviews and meta-analyses of RCTs or observational human studies to inform all different aspects of GIOP prevention and treatment. In case multiple meta-analyses were available, we gave priority to network meta-analyses and meta-analyses that included the most recent studies. However, a preliminary search showed that a recent meta-analysis was lacking for several subtopics. When a recent meta-analysis was unavailable, evidence prior to 2005 was also considered, and we searched for published RCTs or relevant observational human studies, depending upon the research question.

The inclusion criteria were defined in terms of PICOS (44): population, intervention, comparator, outcome and study type. The population was defined as glucocorticoid-treated adults. Each guideline subtopic (*e.g.* calcium and/or vitamin D, monitoring, exercise *etc.*) was considered as an intervention. Interventions (*e.g.* drugs) not available in Belgium, studies in children, or meta-analyses focusing exclusively on different populations (*e.g.* Asian countries) were excluded. Placebo, open-label or active-controlled comparators were allowed. Priority was given to fracture outcomes and falls, but in their absence, BMD and biochemical parameters were also included.

Keywords in our search included “glucocorticoids”, “steroid-induced” or “corticosteroids”, “osteoporosis”, “fracture” or (“bone” AND “density”), and keywords related to each of the subtopics of this consensus document. We also searched for guidelines from other scientific societies, and extracted relevant systematic review, meta-analyses, RCTs or observational studies from the reference lists. Finally, each of the authors was allowed to provide additional references.

We used the simplified GRADE system to indicate the strength of recommendations and the quality of the supporting evidence (**Table 1**) (45).

**Table 1:** Simplified GRADE approach.

STRENGTH OF RECOMMENDATION	
**Strong recommendation**	Wording: “we recommend”
	A strong recommendation implies that the benefits clearly outweigh the risks and burdens (or *vice versa*), and that virtually all informed patients would choose to follow this recommendation.
**Weak recommendation**	Wording: “we suggest” or “consider”
	A weak recommendation implies that the benefits, risks and burdens are closer together or uncertain, and that patients are likely to make different informed choices based on their individual values and preferences.
**No recommendation**	Evidence is currently insufficient to balance the benefits, risks and burdens of an intervention.
**QUALITY OF EVIDENCE**	
**High**	- Clear evidence from a large meta-analysis or at least one large (N ≥ 1000), methodologically sound randomized controlled trial, or - Very large (N ≥ 10000) observational studies showing large and consistent effects
**Moderate**	- Meta-analysis yielding significant effects, but with few participants (N < 1000), some heterogeneity or limited generalizability, - Randomized trial(s) with small sample size, moderate risk of bias or limited generalizability, or - Large (N ≥ 1000) observational studies showing consistent effects
**Low**	- Most non-randomized studies, poor-quality randomized trials, small observational studies or expert opinion

The Belgian Bone Club (BBC) is a multidisciplinary national scientific society established in 1987, devoted to promoting understanding of bone disorders in general and osteoporosis in particular. All co-authors were asked to review and shape recommendations pertaining to one particular subtopic, but all co-authors revised the different subtopics in consecutive revisions. Furthermore, all authors discussed and unanimously agreed upon the grading of the recommendations and the assessment of the quality of the supporting evidence. Following keynote lectures on clinical trials and guidelines by two international experts, these recommendations were presented to membership at the 2021 BBC Clinical Update Symposium and refined further after plenary discussions.

## 3 Results

More than 150 systematic reviews and meta-analyses were identified and included in this work. Ten strong and ten weak recommendations were formulated, based on high quality (2 recommendations), moderate quality (7 recommendations) or low quality evidence (11 recommendations). The different subtopics of this review and their corresponding recommendations (with the strength of the recommendation and quality assessment of the underlying evidence), are summarized in **Table 2** and discussed below in more detail.

**Table 2:** Summary of the BBC recommendations for GIOP in adults.

TOPIC	RECOMMENDATION
**Appropriate use of glucocorticoids**	1. To prevent GIOP and other complications of long-term glucocorticoid use, we recommend using the lowest effective dose for the shortest amount of time (*strong recommendation, high-quality evidence*).
	2. We recommend administering glucocorticoids locally rather than systemically when this may be equally effective (*strong recommendation, moderate quality evidence*).
	3. We suggest early consideration of equally safe and effective glucocorticoid-sparing alternatives (*weak recommendation, low-quality evidence*).
	4. We suggest that policymakers, scientific societies and care organizations at all levels prioritize implementation of strategies to close the treatment gap in GIOP (*weak recommendation, low-quality evidence*).
**Non-pharmacological management**	5. We recommend educating all glucocorticoid-treated patients regarding the risk of osteoporosis and fractures and strategies to avoid those risks (*strong recommendation, low-quality evidence*).
	6. We recommend eating a balanced diet, exercising regularly, keeping weight within the recommended range, stopping smoking, and preventing alcohol abuse as important lifestyle measures for all adults, and *a fortiori* for patients receiving glucocorticoids (*strong recommendation, low-quality evidence*).
	7. We suggest a falls risk assessment in older adult glucocorticoid users, and implementation of evidence-based strategies to reduce the risk of falling in people at increased risk (*weak recommendation, low-quality evidence*).
	8. Given the lack of evidence to support symptomatic or functional benefit, and possible risk of complications, we recommend against routine vertebroplasty or kyphoplasty in GIOP patients with vertebral compression fractures (*strong recommendation, moderate quality evidence*).
**Laboratory evaluation**	9. We suggest a basic biochemical evaluation for other secondary causes of osteoporosis in all glucocorticoid users (preferably before the start of glucocorticoid therapy), regardless of their fracture risk (*weak recommendation, low-quality evidence*).
**Fracture risk assessment and treatment eligibility**	10. We recommend fracture risk assessment using clinical risk factors, vertebral fracture assessment and DXA to guide treatment decisions in GIOP (*strong recommendation, moderate quality evidence*).
	11. We recommend early GIOP prevention in post-menopausal women and men or women aged > 40 years with very high fracture risk (prior vertebral or hip fracture, T-score ≤ -2.5), and for those at high fracture risk (increased FRAX^®^ score, T-score ≤-1.5 or use of ≥ 7.5 mg prednisolone equivalents/day) (*strong recommendation, moderate quality evidence*).
	12. We suggest considering early GIOP prevention in pre-menopausal women or men aged < 40 years with high to very high fracture risk (*weak recommendation, low-quality evidence*).
**Calcium and vitamin D**	13. In adults at risk of bone loss or fractures (which includes glucocorticoid users), we recommend an optimal total daily calcium intake from 1200 to 2000 mg (preferably from dairy or other nutritional sources), together with vitamin D supplements of 800 – 1000 IU/day to achieve a total 25-hydroxyvitamin D target level of 50-125 nmol/L (20-50 ng/mL) (*strong recommendation, low-quality evidence*).
**Antiresorptive and bone anabolic drugs**	14. We recommend alendronate, risedronate, zoledronate, denosumab or teriparatide for GIOP prevention (*strong recommendation, high-quality evidence*).
	15. We suggest considering oral bisphosphonates in GIOP patients at high fracture risk, and teriparatide, denosumab or zoledronate in GIOP patients at very high fracture risk. Nevertheless, we suggest tailoring the choice between treatments not only according to the risk of fractures, but also according to contraindications, patient preference, cost and the possibility of poor compliance (*weak recommendation, moderate quality evidence*).
**Follow-up and monitoring**	16. We suggest that follow-up in GIOP should focus on adherence to GIOP preventive and treatment measures, and fracture risk re-evaluation at each visit (*weak recommendation, low-quality evidence*).
	17. We suggest BMD monitoring by DXA and vertebral fracture identification one year after glucocorticoid initiation. Thereafter, individualized monitoring intervals might be considered. Extended femur scans might be considered at the time of DXA imaging in glucocorticoid users on long-term (≥ 3 years) antiresorptive therapy (*weak recommendation, low-quality evidence*).
	18. Trabecular bone score (TBS) might be considered during GIOP monitoring, however, the evidence is currently insufficient to alter treatment based on TBS alone (*weak recommendation, low-quality evidence*).
	19. BTMs may be considered for monitoring treatment with anti-resorptive or osteoanabolic drugs in GIOP (*weak recommendation, moderate quality evidence*).
	20. When glucocorticoids are discontinued, we recommend a re-evaluation of fracture risk to guide the decision to continue or stop GIOP prevention (*strong recommendation, moderate quality evidence*).

### 3.1 Appropriate Use of Glucocorticoids

- *Recommendation 1: To prevent GIOP and other complications of long-term glucocorticoid use, we recommend using the lowest effective dose for the shortest amount of time (strong recommendation, high-quality evidence)*.

Meta-analyses of RCTs and very large observational studies consistently show that the association of glucocorticoid excess with fractures, BMD decline and increased bone turnover is dose- and duration-dependent, while tapering or stopping glucocorticoids reduces these risks (5, 9, 12, 15, 46, 47). Moreover, high-dose glucocorticoids may result in less fracture risk when given in short course rather than chronically (15, 48). In COPD for example, shorter glucocorticoid pulses are nowadays preferred over longer courses, due to similar effectiveness and the possibility of less cumulative toxicity (49). Glucocorticoid stewardship in general involves prescribing the lowest effective dose for the shortest possible time (50).

Prolonged courses of high glucocorticoid doses remain a cornerstone of therapy in many conditions *e.g.* RA, polymyalgia rheumatica/giant cell arteritis, or immune-mediated hemolytic anemia/thrombocytopenia. Some of these patients are considered glucocorticoid-dependent but can still be tapered after prolonged follow-up. Others may suffer tertiary adrenal insufficiency, which may also be amenable to slow glucocorticoid weaning. In some progressive conditions such as Duchenne muscular dystrophy, glucocorticoids are the standard of care, although they contribute to the high fracture burden in these younger patients (51). Despite the necessity for prolonged glucocorticoid usage in these conditions, attention to GIOP prevention remains poor, particularly in hematological conditions such as immune thrombocytopenia, autoimmune hemolytic anemias or lymphomas (52–54).

There is reassuring evidence that long-term low-dose hydrocortisone replacement therapy in Addison’s disease is safe (55). However, glucocorticoid doses are often above the physiological range in patients with adrenal insufficiency (46) and fractures rates appear to be increased in this context (56). The use of prednisone, prednisolone or dexamethasone rather than hydrocortisone, or higher hydrocortisone doses, have been associated with lower BMD, although the available evidence is scant and heterogeneous (55) (57–59). There is also low-quality evidence that primary hyperaldosteronism might increase the risk of GIOP (60, 61).

- *Recommendation 2: We recommend administering glucocorticoids locally rather than systemically when this may be equally effective (strong recommendation, moderate quality evidence)*.

Local glucocorticoid therapy remains an underused yet feasible strategy in dermatological conditions, respiratory diseases (intranasal or inhaled corticosteroids), acute non-infectious mono-arthritis (intra-articular injection) or IBD (delayed-release oral formulations). Nevertheless, some systemic glucocorticoid exposure remains with all of these routes, which are associated with a non-negligible risk of GIOP in chronic users, mainly at high doses (47) (62–64).

Systemic glucocorticoids increase the risk of GIOP and fractures in asthma, while the effect of high doses of inhaled corticosteroids remains equivocal (24) (65, 66). In COPD, a meta-analysis of RCTs and observational studies showed that inhalation corticosteroids are associated with a significant but modest dose-dependent increase in fracture risk (67) although RCTs alone show no clear effect on BMD (68). High-dose cumulative corticosteroid exposure from epidural injections is associated with lower BMD and increased vertebral fracture risk, particularly in post-menopausal women not receiving concomitant anti-osteoporosis medication (69). In early active RA, one RCT showed that intra-articular glucocorticoids were associated with bone loss, which could be prevented by alendronate (70). Glucocorticoid-naïve Crohn’s disease patients showed less bone loss with oral budesonide (which is available as a delayed release formulation and has extensive first-pass hepatic metabolism) than with prednisolone in one RCT (71). However, this may be associated with lower efficacy (72) and budesonide showed some evidence of bone loss in primary biliary cirrhosis (73). A recent nationwide retrospective cohort study showed that potent topical corticosteroids also slightly increase the risk of osteoporotic fractures (64) but whether this risk is lower than for oral glucocorticoids remains unknown. For other routes of administration however, the risk of GIOP remains poorly defined (74). Local administration may carry a risk of local side-effects *e.g.* joint infection in case of breached injection sterility. Therefore, the benefits and risks of local *vs*. systemic glucocorticoids need to be carefully balanced according to the needs and the preferences of each specific patient.

- *Recommendation 3: We suggest early consideration of equally safe and effective glucocorticoid-sparing alternatives (weak recommendation, low-quality evidence).*

Glucocorticoid-sparing regimens are increasingly popular in organ transplantation (75) psoriasis, IBD and rheumatological conditions. In general, however, high-quality evidence that glucocorticoid-sparing therapies reduce fracture risk in these patients remains lacking (76). In a meta-analysis in RA patients, tumor necrosis factor inhibitors showed no significant effect on hip and spine BMD compared to placebo, whereas glucocorticoids impaired spine BMD compared to placebo (26). Direct comparisons remain however missing.

Alternative immunosuppressants also have side effects, and it is often easier, more evidence-based and cheaper to apply GIOP prevention or treatment rather than switching to biologicals solely because of osteoporosis risk. Cyclosporine and tacrolimus are also associated with bone loss, while azathioprine and mycophenolic acid are not (20). Methotrexate is only very rarely associated with osteopathy, mainly at excessive doses or due to drug interactions (77). While the mammalian target of rapamycin (mTOR) pathway plays an important role in bone metabolism, the clinical effects of mTOR inhibitors on osteoporosis or fracture risk remain poorly understood (20).

- *Recommendation 4: We suggest that policymakers, scientific societies and care organizations at all levels prioritize implementation of strategies to close the treatment gap in GIOP (weak recommendation, low-quality evidence)*.

Despite the frequency and the severe complications of GIOP, and the existence of clear guidelines and evidence-based fracture reduction strategies, considerable underdiagnosis and undertreatment of GIOP remains (78). A systematic review of observational studies showed that < 40% of chronic glucocorticoid users received BMD testing or osteoporosis medications (79). Although the use of bisphosphonates in GIOP has increased since their introduction, there is little evidence of improvement in GIOP prevention over time (80). Although there is strong evidence of a treatment gap in GIOP, the strength of the recommendation was judged “weak” because of a lack of strategies with demonstrated efficacy to close the gap.

One RCT showed that pharmacist feedback did not increase the uptake of bisphosphonates for GIOP prevention. Although a beneficial effect was observed in the subgroups of men and patients ≥ 70 years, the prescription rate in these populations still only increased from 5% in the control group to 13% in the intervention group (81). Similar RCTs of pharmacist-led interventions confirm positive trends but with a very large remaining treatment gap (82, 83). Nevertheless, system-based and electronic medical record-based interventions (84) may be more successful than patient or provider education alone (85). Some studies have reported improved compliance with GIOP guidelines following a multidisciplinary quality improvement initiative (86, 87). However, another trial of a web-based quality improvement program found no significant effect (88). One systematic review found that no GIOP care quality improvement interventions produced robust changes, with overall adherence remaining low (85).

Notably, implementation of multifaceted interventions to improve GIOP prevention might be cost-effective if they succeed in promoting uptake of generic bisphosphonates in a large proportion of high-risk patients (89). Barriers in fracture prevention include cost of therapy, time and cost required for diagnosis, concerns about medication side-effects, and lack of clarity regarding the responsibility to undertake GIOP prevention (90). We believe GIOP prevention is the responsibility of every physician prescribing glucocorticoids. In case of doubt however, asking advice from, or referring to a specialist in osteoporosis is encouraged.

### 3.2 Non-Pharmacological Management

- *Recommendation 5: We recommend educating all glucocorticoid-treated patients regarding the risk of osteoporosis and fractures and strategies to avoid those risks (strong recommendation, low-quality evidence).*

In line with the BBC guidelines on post-menopausal osteoporosis (44), we highly recommend patient education as likely being beneficial from a patient perspective, although there is no direct supporting evidence. A discussion on GIOP is particularly advised with long-term (≥ 3 months) glucocorticoid users, while reassurance may be offered to short-term (< 3 months), low-dose (< 5 mg/day) users at low fracture risk. Patients can be reassured that osteoporosis is generally not a contraindication for glucocorticoid therapy if they have an evidence-based indication for such a therapy, provided that the risk of GIOP is systematically assessed and appropriately treated.

- *Recommendation 6: We recommend eating a healthy diet, exercising regularly, keeping weight within the recommended range, stopping smoking, and preventing alcohol abuse as important lifestyle measures for all adults, and a fortiori for patients receiving glucocorticoids (strong recommendation, low-quality evidence).*
- *Recommendation 7: We suggest a falls risk assessment in older adult glucocorticoid users, and implementation of evidence-based strategies to reduce the risk of falling in people at increased risk (weak recommendation, low-quality evidence).*

Our previous GIOP guidelines recommended that non-pharmacological treatment (primarily nutrition and exercise) should be considered (43). More than fifteen years later, most international GIOP guidelines still recommend lifestyle as an important general management strategy, despite lack of evidence (91).

Glucocorticoid excess causes detrimental effects not only on bone but also on muscle, cartilage and fat mass, all of which might be mitigated by a healthy diet and regular physical exercise. It should be emphasized, however, that only indirect evidence is available, based on data in postmenopausal osteoporosis or from preclinical or clinical studies of limited quality and quantity in GIOP. Still, a healthy lifestyle has numerous other beneficial effects on health and quality of life. When it comes to physical exercise, the focus should be on body balance, impact training, and strength and resistance training tailored to the needs and abilities of each patient, as recommended by the BBC guidelines on post-menopausal osteoporosis (44).

Glucocorticoids might also increase the risk of falls. In a population with median age 60 years, Van Staa *et al.* reported that falls incidence increased from 1.6 to 2.8 per 100 person-years in the first 3 months of glucocorticoid use, and decreased to baseline fairly rapidly after discontinuation (8). Clinical trials on fall prevention in glucocorticoid users remain however lacking. We suggest falls risk screening in adults aged 65 years and older, although this is based on expert opinion. Even in adults in midlife, very high glucocorticoid doses may lead to significant muscle atrophy and risk of falls.

Simple assessments are available and recommended to screen for muscle weakness and/or falls risk in GIOP patients. These include identification of clinical risk factors for falls, such as history of falls in the previous year, mobility limitations, visual impairments, neuromuscular diseases, advanced age, polypharmacy and sarcopenia. Clinical assessment of falls risk involves observing gait and mobility, or more formal practical tests such as the sit-to-stand test, four-stage balance test, timed up and go test, gait speed, Tinetti assessment of balance and gait, *etc.* Multifactorial and exercise interventions have shown greatest promise in reducing falls risk in older adults in general (92).

- *Recommendation 8: Given the lack of evidence to support symptomatic or functional benefit, and possible risk of complications, we recommend against routine vertebroplasty or kyphoplasty in GIOP patients with vertebral compression fractures (strong recommendation, moderate quality evidence).*

Given the frequency of vertebral compression fractures in GIOP, it is relevant to note that a Cochrane systematic review found high to moderate quality evidence that vertebroplasty has no important benefit in terms of pain, disability or quality of life in routine practice when compared with a sham procedure (93, 94). Furthermore, a systematic review found an increased risk of vertebral fractures following vertebroplasty in patients with low bone mineral density as well as in glucocorticoid users (odds ratio 2.632; 95% CI 1.399 to 4.950) (95). Still, patients with vertebral fractures, particularly with posterior displacement, should be educated regarding and monitored for symptoms of spinal cord compression, and be considered for surgical referral. In individual patients with exceptional pain refractory to optimal analgesia however, referral to a bone specialist might be considered to discuss the potential benefit in terms of pain relief *vs*. the risk of complications (including additional vertebral fractures).

### 3.3 Laboratory Evaluation

- *Recommendation 9: We suggest a basic biochemical evaluation for other secondary causes of osteoporosis in all glucocorticoid users (preferably before the start of glucocorticoid therapy), regardless of their fracture risk (weak recommendation, low-quality evidence).*

We suggest a basic biochemical evaluation, even before the start of glucocorticoids, to inform osteoporosis treatment and to identify possible additional secondary causes of osteoporosis. Suggested basic and optional additional laboratory tests are listed in **Table 3** (96). The suggested laboratory investigation is similar as in post-menopausal osteoporosis (44). There are only observational studies reporting on biochemical testing to identify secondary causes of osteoporosis (44). In GIOP specifically, even those studies appeared lacking in our literature search. Additional tests to identify or guide management of other side effects of glucocorticoids (*e.g.* fasting glucose, hemoglobin A1c, lipid panel, *etc.*) may simultaneously be considered.

**Table 3:** Recommended laboratory tests in the evaluation of glucocorticoid-induced osteoporosis.

**Basic laboratory tests**	Complete blood count
	Creatinine and estimated glomerular filtration
	Plasma calcium and phosphate, parathyroid hormone
	25-hydroxyvitamin D
	Alkaline phosphatase + liver function tests
	Serum protein electrophoresis
	Thyroid stimulating hormone
**Optional tests** (in case of abnormalities or on clinical indication)	24h urinary calcium excretion
	Total and free testosterone (in men), estradiol and gonadotropins (in non-menstruating premenopausal women not taking contraceptives), sex hormone-binding globulin
	Anti-tissue transglutaminase antibody

Currently available circulating biomarkers are not useful to distinguish primary from secondary osteoporosis in general, or to diagnose GIOP in particular. Serum biomarkers may however be useful for osteoporosis treatment monitoring (44). Two bone turnover markers (BTMs), namely procollagen type I N-propeptide (PINP), a bone formation marker, and C-terminal cross-linking telopeptide of collagen (β-CTX), a bone resorption marker, are currently preferred for osteoporosis treatment monitoring (97, 98). One bone formation marker (*e.g.* P1NP or bone-specific alkaline phosphatase) and one bone resorption marker (e.g. β-CTX or TRAP-5b) can be prescribed per request and are refunded in Belgium, in case of (suspected) metabolic bone disease. Yet, their interpretation is rather difficult due to preanalytical (circadian rhythm, influence of food intake) and analytical factors (lack of standardization). Blood samples should preferably be obtained in the morning in the fasted stated (especially for β-CTX). In patients with renal insufficiency, bone-specific alkaline phosphatase, intact P1NP or TRAP-5b should be preferred over total P1NP or β-CTX. Due to the lack of standardization of β-CTX assays we recommend that patients should be followed by the same laboratory and that laboratories performing bone turnover measurement mention the method used on their results protocols (99, 100). Of note, BTM concentrations increase and may remain elevated up to one year after a fracture. Furthermore, BTMs may be affected not only by initiation of glucocorticoids, but also by the underlying disease (101).

Biomarkers are useful in research to help understand the mechanisms of bone loss in GIOP, and they have been used frequently in clinical trials. The most studied BTM in GIOP is osteocalcin. After initiation of glucocorticoids in healthy volunteers, the bone formation markers osteocalcin and PINP both rapidly decrease (102, 103) and the larger the dose given, the larger the decline (104). Bone resorption markers (such as urinary N-terminal cross-linked telopeptide of type 1 collagen) on the other hand slightly increase in young healthy volunteers (101, 104), suggesting a dissociation between bone formation (decreased) and resorption (increased). In post-menopausal women, bone resorption markers may decrease early after experimental low-dose glucocorticoid administration, likely due to a dominant effect on reduced bone formation (105). Interestingly, osteocalcin concentrations return to baseline levels after glucocorticoid withdrawal. This also seems to be the case in long-term users who discontinue glucocorticoids.

### 3.4 Fracture Risk Assessment and Treatment Eligibility

- *Recommendation 10: We recommend fracture risk assessment using clinical risk factors, vertebral fracture assessment and DXA to guide treatment decisions in GIOP (strong recommendation, moderate quality evidence).*

Fracture risk in GIOP is associated with clinical risk factors including advanced age, sex, prior fractures, low BMI, dose and duration of glucocorticoid therapy, falls history as well as other traditional risk factors (3, 9, 12, 36). The absolute fracture incidence in GIOP is higher at older ages, whereas the relative increase due to glucocorticoids may become smaller with advancing age (3). For the same BMD, there is no significant difference in glucocorticoid-induced relative fracture risk between men and women (3). Cost-effectiveness of GIOP treatment improves with higher fracture risk in individual glucocorticoid users (106, 107). RCTs of drugs to prevent or treat GIOP have generally selected participants based on DXA T-scores, prior fracture history, or both. Because GIOP is associated with greater fracture risk than post-menopausal osteoporosis for the same age and BMD, there is a rationale for higher T-score cut-offs for treatment in GIOP (3).

The BBC guidelines on post-menopausal osteoporosis recommend fracture risk assessment using calculators like FRAX^®^ or Garvan (44). FRAX^®^ is the most commonly used tool in Belgium, since calculation of the FRAX^®^ score at the time of bone densitometry is mandated for DXA reimbursement. Previous or current (/anticipated) exposure to ≥ 5 mg prednisolone/day (or equivalent) for 3 months or longer is a BMD-independent fracture risk factor in FRAX^®^ (3), whereas glucocorticoids are not accommodated in the Garvan nomogram.

FRAX^®^ and a number of other algorithms lack the ability to provide estimates for patients < 40 years, while many glucocorticoid users are this young. However, fracture incidence in glucocorticoid users below 40 years is very low (9), and treatment below this age is unlikely to be cost-effective using a short time horizon (106). Another limitation of FRAX^®^ is that use of systemic glucocorticoids is entered as a dichotomous risk factor (yes/no), assuming an average risk. Current and cumulative dose (*e.g.* from intermittent courses) are not taken into account. Under certain assumptions, formulas to adjust conventional FRAX^®^ estimates of hip and major osteoporotic fracture probabilities according to the glucocorticoid dose have been published (**Table 4**) (108). Still, clinical judgement should assume even higher risk for very high glucocorticoid doses, even when given for < 3 months duration (109). Of note, national FRAX^®^ models may require recalibration, as recently shown by a large Belgian population-based study (110, 111).

**Table 4:** Simplified algorithm with percentage adjustment of 10-year probabilities of a hip fracture or a major osteoporotic fracture by age according to dose of glucocorticoids (108).

	GC dose	Age					
**Hip fracture**		40 yr	50 yr	60 yr	70 yr	80 yr	90 yr
Low	< 2.5	-40%	-40%	-40%	-40%	-30%	-30%
Medium	2.5-7.5	Referent group					
High	≥7.5	+25%	+25%	+25%	+20%	+10%	+10%
**MOF**							
Low	< 2.5	-20%	-20%	-15%	-20%	-20%	-20%
Medium	2.5-7.5	Referent group					
High	≥7.5	+20%	+20%	+15%	+15%	+10%	+10%

MOF, Major osteoporotic fracture; GC, glucocorticoid dose, expressed as mg prednisolone equivalents/day.

Both the American College of Rheumatology (ACR) guidelines as well as the International Osteoporosis Foundation (IOF)/European Calcified Tissue Society (ECTS) guidelines on GIOP advise a baseline DXA at the onset of glucocorticoid therapy to better discriminate high-risk from lower-risk patients (91, 96). Vertebral fracture assessment (VFA) at the time of DXA (or spine X-rays) may identify prevalent vertebral fractures, which strongly influences fracture risk and treatment recommendations. The BBC suggests that trabecular bone score (TBS), which can be used to adjust FRAX^®^ scores, may be used to fine-tune baseline and especially follow-up fracture risk assessment (see *Section 3.7.* below) (112).

In Belgium, parenteral osteoporosis drugs are generally reimbursed only in people with prior radiographic vertebral fractures, hip fractures or DXA T-scores < -2.5 at the lumbar spine or hip. Thus, DXA and spinal X-rays may be used not only for fracture risk assessment, but also for reimbursement purposes, to reduce out-of-pocket expenses for patients. However, initiation of pharmacotherapy *e.g.* oral bisphosphonates, should not be delayed to obtain a bone densitometry (113). Moreover, in patients with prior vertebral or hip fractures, there may be no need to obtain a DXA, since they often qualify for treatment and reimbursement anyway.

Given that fracture risk increases in GIOP well before any changes are observed on DXA, there is a clear need for additional ways to improve fracture risk assessment in GIOP. High-resolution quantitative computed tomography (114–118) or reference point indentation (119) may be more sensitive that DXA to detect declines in certain bone qualities, although only proof of principle studies are available (120).

- *Recommendation 11: We recommend early GIOP prevention in post-menopausal women and men or women aged > 40 years with very high fracture risk (prior vertebral or hip fracture, T-score ≤ -2.5), and for those at high fracture risk (increased FRAX^®^ score, T-score ≤-1.5 or use of ≥ 7.5 mg prednisolone equivalents/day) (strong recommendation, moderate quality evidence).*
- *Recommendation 12: We suggest considering early GIOP prevention in pre-menopausal women or men aged < 40 years with high to very high fracture risk (weak recommendation, low-quality evidence).*

Since fracture risk increases shortly after initiating glucocorticoid therapy, most guidelines emphasize initiation of bone-sparing drugs in eligible patients as early as possible. In general, since prednisolone doses in the range of 2.5-7.5 mg prednisolone are associated with increased fracture risk within 3 months (8, 9) GIOP prevention might be considered in all patients committed or exposed to such glucocorticoid doses. However, as in the general and other clinical populations, patients with the highest risk for fracture are the ones most likely to benefit from non-pharmacological and pharmacological fracture prevention (lowest number needed to treat). Likewise, the cost-effectiveness of GIOP prevention is sensitive to intervention cost, willingness to pay, and fracture risk factors such as older age, lower BMD, higher doses of glucocorticoids and the number of prior fractures (106, 107) (121–124). Thus, despite limitations in our accuracy to predict fracture risk, most guidelines recommend different treatment strategies for patients estimated to be at low, high or very high fracture risk (91). Fracture risk is usually classified in different GIOP guidelines according to DXA T-scores, FRAX^®^ scores, or both (125).

The IOF–ECTS working group on GIOP suggests considering an estimated 10-year fracture risk of 20% as an intervention threshold (96). From a practical point of view, this would imply that virtually all glucocorticoid-treated patients ≥ 70 years and/or with a previous or incident fragility fracture, and all postmenopausal women and men ≥ 50 years on a high dose of glucocorticoids (≥ 7.5 mg prednisolone/day) or with a T-score ≤ -1.5 would be considered eligible for treatment (**Table 5**) (96). This coincides more or less with cost-effectiveness thresholds, assuming that drugs are similarly effective in GIOP as they are in post-menopausal osteoporosis (123). This framework proposes tailoring recommendations based on country-specific models. Unfortunately, no official willingness to pay threshold exists in Belgium. Our recent national guidelines on post-menopausal osteoporosis suggest a treatment threshold of ≥ 20% for 10-year osteoporotic fracture risk, ≥ 3% for hip fracture risk in individuals aged < 70 years, and ≥ 5% in those aged ≥ 70 years (44). For consistency, we suggest to extend these cut-offs to GIOP in Belgium. We emphasize that the BMD thresholds for the high and very high risk categories in these recommendations pertaining to GIOP, are higher than in our guidelines for postmenopausal osteoporosis (44).

**Table 5:** Fracture risk groups eligible for bone-sparing drugs in IOF-ECTS (96) and ACR guidelines (91) on GIOP.

IOF-ECTS		American College of Rheumatology	
Premenopausal women and men < 50 years	Post-menopausal women and men ≥50 years	Adults < 40 years of age	Adults ≥ 40 years of age
Previous fracture	Previous fracture	Prior osteoporotic fracture(s)	Prior osteoporotic fracture(s)
	Age ≥70 years		
	Glucocorticoid daily dose ≥7.5 mg prednisolone		
	BMD T-score ≤ -1.5	BMD Z-score < -3.0 or ≥10% BMD loss/year AND continuing ≥ 7.5 mg/day, ≥ 6 months	BMD T-score ≤ -2.5 in post-menopausal women or men ≥50 years
	FRAX^®^ 10-year risk of ≥ country-specific threshold		FRAX^®^ 10-year risk of ≥ 10% for major osteoporotic fractures, ≥ 1% for hip fractures
		Very high glucocorticoid dose*, age ≥ 30 years	Very high glucocorticoid dose*

*Very high glucocorticoid dose = initial dose ≥ 30 mg/day and cumulative annual dose > 5g (prednisolone equivalents).

More recently, the ACR guidelines (91) suggest three operational fracture risk categories for glucocorticoid-treated patients: low, moderate and high fracture risk (see **Table 5**). Categorization is determined by patient’s age (< or ≥ 40 years), prior osteoporotic fractures, T-score at the lumbar spine or hip (Z-score if < 40 years), FRAX^®^ score (if ≥ 40 years), and glucocorticoid dose. In the end, there are many similarities between the American and international/European guidelines.

Another important disease and risk modifier to consider is gonadal status, even more as high-dose glucocorticoid therapy will compromise hypothalamic-pituitary-gonadal function in both men and women. Thus, in premenopausal women who become hypogonadal some form of hormone replacement therapy may be considered. In younger men who develop hypogonadism and are symptomatic, testosterone replacement therapy may be considered. However, either hormone therapy should be considered independent of bone health, since there is insufficient evidence to support sex hormone replacement therapy for GIOP prevention alone (126).

In most patients receiving glucocorticoid replacement therapy for adrenal insufficiency, daily glucocorticoid doses are less than the equivalent of 7.5 mg prednisolone but still often supraphysiologic. Moreover, glucocorticoid treatment in patients with primary adrenal insufficiency is lifelong, and in patients with secondary or tertiary adrenal insufficiency, there might have been detrimental musculoskeletal effects of the primary disease or higher initial iatrogenic glucocorticoid doses. As such, despite good evidence, we suggest to apply the same risk categorization and treatment considerations as in other glucocorticoid-treated patients.

Solid organ and bone marrow transplantation are associated with even higher bone loss than GIOP, and high glucocorticoid doses are still often used (127). The pathophysiology of osteoporosis in organ transplantation extends beyond GIOP alone (75). Bisphosphonates have been shown to improve BMD (128–130) and possibly reduce fracture risk in transplant recipients (131), yet the certainty of the evidence is low. Nevertheless, we suggest extending our recommendations for GIOP to glucocorticoid users who are candidates for or recipients of organ transplants (75).

### 3.5 Calcium and Vitamin D

- *Recommendation 13: In adults at risk of bone loss or fractures (which includes glucocorticoid users), we recommend an optimal total daily calcium intake from 1200 to 2000 mg (preferably from dairy or other nutritional sources), together with vitamin D supplements of 800 – 1000 IU/day to achieve a total 25-hydroxyvitamin D target level of 50-125 nmol/L (20-50 ng/mL) (strong recommendation, low-quality evidence).*

The pathophysiology of GIOP involves decreased intestinal calcium absorption and increased urinary calcium loss (13). Guidelines in osteoporosis in general and GIOP in particular recommend adequate nutritional calcium intake (preferably from dairy sources) and adequate vitamin D status (44). Supplements of calcium and/or vitamin D should be used if these goals are not met.

Meta-analyses show that bone loss at the lumbar spine, hip and forearm is prevented by calcium and vitamin D supplements in glucocorticoid-treated patients (132). A Cochrane meta-analyses concluded that calcium and vitamin D should therefore be initiated prophylactically in all glucocorticoid users (133). However, evidence on fracture outcomes as well as falls remains lacking, the number of participants in these meta-analyses was very low, different types of vitamin D supplements or analogues were used, and evidence concerning optimal doses or target levels in GIOP remains lacking. Therefore, we judged the quality of evidence to be low.

In analogy to recommendations on post-menopausal osteoporosis, many GIOP guidelines recommend doses of calcium and vitamin D between 1,000 to 1,200 mg/day and 600 to 800 IU/day to achieve 25-hydroxyvitamin D target serum levels, respectively (91). Higher doses are not more effective (except in patients with severe deficiency) and several RCTs suggest that intermittent high-dose vitamin D boluses may even increase the risk of falls, bone loss and fractures (134–136). An upper target level of 125 nmol/L (50 ng/ml) is suggested by the BBC guidelines on post-menopausal osteoporosis (44).

There is moderate quality evidence that active vitamin D analogues maintain BMD and reduce vertebral fracture risk in GIOP, and more so than conventional calcium- and vitamin D supplements (132, 137, 138). However, the use of active vitamin D analogues is not recommended, because bisphosphonates or other bone drugs are more effective (138), and the therapeutic window is narrower than with conventional calcium- and vitamin D-supplements.

Likewise, several small randomized trials with calcifediol have reported increased BMD (139) and lower vertebral fracture risk in GIOP or solid organ transplant recipients (140, 141). Nevertheless, the evidence is insufficient to recommend the routine use of calcifediol, which is more expensive and not necessarily better than cholecalciferol or ergocalciferol in combination with bone drugs such as bisphosphonates.

Calcium- and vitamin D-supplements at excessive doses may be associated with an increased risk of hypercalciuria (142, 143), which is of particular concern in GIOP. Some meta-analyses suggest that calcium supplements are associated with cardiovascular adverse events, although these findings remain quite controversial and unconfirmed by other studies (44). Calcium- and vitamin D supplements are unfortunately not reimbursed in Belgium, even though they may be cost-effective in GIOP (144).

Importantly, calcium- and vitamin D-supplements alone are insufficient in many patients, who require combined therapy with bone-sparing drugs. In randomized trials, GIOP patients receiving calcium with or without vitamin D supplements still lost 1-4% lumbar spine BMD over one year (145, 146). Patients at high to very high fracture risk still more often receive calcium and vitamin D, rather than the combination of bisphosphonates with calcium and vitamin D (147). In other words, calcium- and vitamin D-supplements are useful but should not be an excuse not to prescribe bisphosphonates or other bone-targeted medications (148).

### 3.6 Antiresorptive and Bone Anabolic Drugs

- *Recommendation 14: We recommend alendronate, risedronate, zoledronate, denosumab or teriparatide for GIOP prevention (strong recommendation, high-quality evidence).*
- *Recommendation 15: We suggest considering oral bisphosphonates in GIOP patients at high fracture risk, and teriparatide, denosumab or zoledronate in GIOP patients at very high fracture risk. Nevertheless, we suggest tailoring the choice between treatments not only according to the risk of fractures, but also according to contraindications, patient preference, cost and the possibility of poor compliance (weak recommendation, moderate quality evidence).*

Regulatory approval of drugs to reduce fracture risk in individuals taking glucocorticoids has been based on RCTs showing improvements in BMD. Fractures have been a secondary outcome, and quality of life endpoints remain lacking (149). The duration of most RCTs has been relatively short (mostly 1-3 years) and this, combined with smaller trial populations, reduces the power of the efficacy and safety data. Nevertheless, a Cochrane meta-analysis found high-quality evidence that bisphosphonates (as a class) reduce vertebral fracture risk in GIOP (149). Furthermore, network meta-analyses of RCTs have found moderate quality evidence that alendronate, risedronate, zoledronate, teriparatide and denosumab reduce fracture risk in GIOP (137, 150). For raloxifene and ibandronate, RCTs have reported increased spine and hip BMD (151) but evidence on fracture risk is insufficient (particularly for the key outcome of vertebral fractures) (152). One recent RCT found a significant but small increase in lumbar spine BMD with bazedoxifene compared to placebo in RA patients taking low glucocorticoid doses (153) but this drug is no longer available in Belgium. Still, raloxifene might be considered in post-menopausal women with contraindications to all other drugs, but no contraindication to a selective estrogen receptor modulator (such as increased risk of cardiovascular disease or venous thromboembolism) (91).

There is high-quality evidence that bone-protective agents prevent GIOP (154, 155). Therefore, we strongly recommend initiation of bone-targeted drugs in patients at risk of GIOP as early as possible, preferably at the start of glucocorticoid treatment. Still, it is never too late to initiate GIOP prevention in the large group of untreated long-term glucocorticoid users, since RCTs have demonstrated efficacy in both new and chronic glucocorticoid users (152). In population-based studies, delayed initiation of anti-osteoporosis medications has been associated with increased vertebral and hip fracture risk (156).

Glucocorticoids themselves, as well as the underlying disease, co-morbidities and co-medications, may explain why patients treated for GIOP might be susceptible to side effects. Glucocorticoid therapy is a documented risk factor for osteonecrosis of the jaw (157) and probably also for atypical femoral fractures (158). Meta-analyses have found no significant increase in upper gastrointestinal side effects with oral bisphosphonates in GIOP trials; however, these trials may collectively have been underpowered (154). A recent very large population-based cohort study found significantly increased fracture risk in RA patients co-prescribed glucocorticoids and proton pump inhibitors, compared to non-use or either drug alone (159).

Most guidelines give preference to oral bisphosphonates as first-line GIOP drugs, because they are easy to use, safe, cheap and cost-effective (91, 106, 107) (121–124). In terms of comparative effectiveness however, network meta-analyses of RCTs in GIOP suggest that teriparatide and denosumab reduce the risk of vertebral fractures more than oral bisphosphonates (152, 160, 161). Individual RCTs in GIOP have shown that teriparatide, denosumab and zoledronate produce greater BMD gains than risedronate (162–164). Furthermore, there is evidence in GIOP that teriparatide reduces vertebral fracture risk more than alendronate (162, 165). Moreover, compliance with osteoporosis drugs is often suboptimal, but may be poorest with oral bisphosphonates. Therefore, we suggest considering teriparatide, denosumab or zoledronate in patients at very high fracture risk (*i.e.* those with prior vertebral or hip fracture and/or a T-score ≤ -2.5). However, these parenteral treatment options are more expensive. Still, even teriparatide may be cost-effective, at least in patients with very high vertebral fracture risk (122). We suggest considering patient preference in treatment choice (166) which may also be influenced by cost and reimbursement. Specifically, teriparatide is the most expensive and most strictly reimbursed GIOP drug in Belgium, which may prevent its use as first-line therapy in most patients.

In summary, we recommend alendronate, risedronate, zoledronate, denosumab and teriparatide as evidence-based treatment options to prevent fractures in GIOP. Raloxifene might be considered if no alternatives are available (*e.g.* due to contraindications). Other treatments mentioned in our previous recommendations from 2006 (ibandronate, calcitonin, strontium ranelate *etc.*) are no longer recommended.

Given the pathophysiological contribution of impaired osteoblast activity in GIOP, the potential for combination therapy (*e.g.* denosumab plus teriparatide (167)) or sequential therapy (168) as well as sclerostin inhibitors to prevent or reverse GIOP merits further investigation. One recent observational study reported similar effect of one year denosumab or romosozumab for treatment of GIOP in RA patients (169).

Of note, women of childbearing potential should be asked about their desire for future pregnancies, counselled regarding the benefits and risks of osteoporosis medications related to pregnancy, and advised to take adequate birth control if appropriate while taking osteoporosis drugs.

#### 3.6.1 Bisphosphonates

Bisphosphonates are the most commonly used drugs in the management of GIOP. Oral alendronate (10 mg daily or 70 mg once weekly) and risedronate (5 mg daily or 35 mg once weekly), and intravenous zoledronate (5 mg once yearly by intravenous infusion) are all approved for this indication (although marketing authorization has not been approved in Europe for all available dosage forms, the BBC does not consider this to be clinically relevant). All improve lumbar spine and hip BMD in patients treated with glucocorticoids (145, 163, 170, 171). Oral bisphosphonates are nowadays generic and cheap. Still, in a comparative effectiveness trial, zoledronate was superior to risedronate to increase lumbar spine BMD (163). For alendronate and risedronate, there is evidence from network meta-analyses that they reduce the rate of vertebral fractures (137, 161). There is moderate quality evidence from meta-analyses (137) as well as real-world observational evidence suggesting that oral bisphosphonates also reduce non-vertebral fractures (172–176). In the pivotal non-inferiority study comparing zoledronate with risedronate, the fracture rate was too low to ascertain anti-fracture effectiveness (163). Given the high fracture risk in GIOP and the proven efficacy of oral bisphosphonates however, placebo-controlled RCTs in GIOP would not have been ethical for newer drugs.

In post-menopausal osteoporosis, a drug holiday may be considered after 5 years of alendronate or 3 annual zoledronate infusions (44). Given their long biological half-life, these drugs continue to reduce fracture risk following their discontinuation, while the risk of adverse effects such as atypical femoral fractures diminishes within months (177). Given the high risk of vertebral fractures however, the concept of drug holiday has not been studied nor recommended in GIOP. Risedronate has a lower affinity for hydroxyapatite and a quicker offset than alendronate or zoledronate, therefore drug holidays have not been studied extensively with risedronate (178). However, there is insufficient data to make recommendations for or against treatment beyond 10 years of oral bisphosphonates or 6 annual zoledronate infusions.

#### 3.6.2 Teripartide

The predominant role of reduced bone formation in GIOP provides a rationale for the use of anabolic agents in its treatment. In an active-comparator RCT the effects of 18 months of treatment with subcutaneous teriparatide, 20 µg/day *vs*. oral alendronate 10 mg/day, were compared in 428 men and women with GIOP. Teriparatide resulted in significantly greater increases in spine and hip BMD than alendronate (162). Although fracture was not a primary end-point of the study, significantly fewer new vertebral fractures occurred in patients treated with teriparatide compared to alendronate (0.6% *vs*. 6.1%; *p* = 0.004) (162). The incidence of non-vertebral fractures was similar in the two treatment groups. Results after 36 months of treatment demonstrated a continued increase in spine and hip BMD in the teriparatide treated group, with superiority over alendronate at the 24-month and 36-month time points (165). A lower incidence of new vertebral fractures was also seen in the teriparatide group at 36 months (1.7% *vs* 7.7%, *p* = 0.007), with a similar incidence of non-vertebral fractures in the two groups. With regard to safety, increased pre-dose serum calcium levels were significantly more common in the teriparatide than alendronate treated group (21% *vs*. 7%), but no other concerns were identified. Although teriparatide does not increase the risk of osteosarcoma in adults (179) it is not recommended for osteoporosis in adult cancer patients (180, 181) due to lack of evidence and concerns that increased remodeling and parathyroid hormone signaling may create a “fertile soil” for the development of bone metastases.

#### 3.6.3 Denosumab

In a phase 3 active-controlled RCT of adults taking ≥ 7.5 mg prednisone or equivalent daily, treatment with denosumab, 60 mg once every 6 months by subcutaneous injection, was associated with a significantly greater increase in lumbar spine and hip BMD compared to risedronate 5 mg daily over a 12 months treatment period (164). This effect was confirmed after 24 months (182) and seen both in patients initiating glucocorticoids and in those on long-term therapy, regardless of age, race, baseline BMD T-score, glucocorticoid dose or menopausal status. HR-pQCT outcomes at the distal radius and tibia were also superior with denosumab in this trial (183). Another head-to-head open-label trial showed superiority of denosumab compared to alendronate in increasing lumbar spine BMD and decreasing BTMs in long-term glucocorticoid users (184).

While denosumab is effective in improving bone mass in glucocorticoid-treated subjects, its discontinuation can be associated with rapid bone loss, high bone turnover and potentially increased risk of so-called rebound-associated multiple vertebral fractures (185). Therefore, it is critical that patients either continue denosumab, or receive follow-up therapy with another anti-resorptive drug such as a bisphosphonate to prevent this complication (185). There are no specific studies on the risk of rebound-associated vertebral fractures in GIOP, but we can hypothesize that the risk may exist (186).

### 3.7 Follow-Up and Monitoring

- *Recommendation 16: We suggest that follow-up in GIOP should focus on adherence to GIOP preventive and treatment measures, and fracture risk re-evaluation at each visit (weak recommendation, low-quality evidence).*

We suggest that history taking and clinical examination focus on recent falls and incident fractures, smoking and alcohol use, muscle strength and balance, BMI, monitoring of height, back pain and kyphosis, and treatment-related adverse events (96). Symptoms of hip or knee pain might precede atypical femoral fractures.

- *Recommendation 17: We suggest BMD monitoring by DXA and vertebral fracture identification one year after glucocorticoid initiation. Thereafter, individualized monitoring intervals might be considered. Extended femur scans might be considered at the time of DXA imaging in glucocorticoid users on long-term (≥ 3 years) antiresorptive therapy (weak recommendation, low-quality evidence).*

Three tools can be used to monitor GIOP in routine clinical practice: BMD measurements by DXA (and fracture risk estimation using updated clinical risk factors), diagnosis of vertebral fractures (by VFA or lateral spine X-ray) and BTMs (see *recommendation 19*).

High glucocorticoid doses cause rapid bone loss, particularly at the lumbar spine and during the first 12 months of treatment (5). In contrast, pharmacotherapy may improve BMD after one year. Thus, both in patients receiving and not receiving bone-active drugs, re-evaluation of BMD may be considered one year after the initiation of glucocorticoids, and every 1 to 5 years thereafter with the shorter interval for those who are taking higher glucocorticoid doses and/or having lower BMD values (91, 187, 188). However, considering the lack of evidence on the clinical value of BMD monitoring in GIOP, we suggest individualized clinical decision making to determine DXA intervals.

VFA (or lateral spine X-rays) to exclude vertebral fractures should be considered at the time of BMD measurement, in case of back pain, or height loss greater than 2 cm since baseline or 4 cm since young adulthood. Improved or stable BMD may be a reason to continue therapy, whereas worsening or stable osteoporosis or identification of (incident or previously unrecognized) vertebral fractures may be an indication to switch drug therapy (see *recommendation 15*).

Some centers have started using extended DXA scanning (including the subtrochanteric region of both femurs) to screen for cortical thickening (“beaking”) or other radiographic features of (subclinical) atypical femoral fractures (189, 190). The International Society for Clinical Densitometry suggests to consider the use of bilateral full-length femur imaging in patients who are currently or have been in the past year on potent antiresorptive therapy (*i.e.*, oral or intravenous bisphosphonate or subcutaneous denosumab therapy) for a cumulative period of 3 or more years, especially those on long-term glucocorticoid therapy (191). However, the evidence is currently insufficient to make definite recommendations or assess the cost-effectivity of such a screening strategy during antiresorptive treatment in general, and in GIOP in particular.

- *Recommendation 18: Trabecular bone score (TBS) might be considered during GIOP monitoring, however, the evidence is currently insufficient to alter treatment based on TBS alone (weak recommendation, low-quality evidence).*

TBS has recently emerged as possibly adding value to the assessment of bone microarchitectural decay during glucocorticoid treatment (192–196). In one RCT in GIOP, TBS showed greater improvement with teriparatide than with alendronate therapy (197). TBS can be considered an ancillary test that can be integrated in the FRAX^®^ algorithm. However, the exact clinical role of TBS in guiding treatment decisions remains unclear.

Although TBS can be considered a promising tool that may even be more sensitive to change than DXA BMD under certain conditions, we suggest considering TBS results together with BMD and clinical risk factors and not in isolation.

- *Recommendation 19: BTMs may be considered for monitoring treatment with anti-resorptive or osteoanabolic drugs in GIOP (weak recommendation, moderate quality evidence).*

Randomized trials in GIOP have shown that teriparatide increases, whereas antiresorptive drugs further reduce bone turnover (165) (198). However, very few studies have evaluated the clinical impact of measuring biomarkers in routine clinical practice. Moreover, specific real-world studies in GIOP are lacking. Thus, whether it improves adherence to osteoporosis drugs or skeletal outcomes in GIOP remains unknown. Monitoring treatment-related changes in BTMs is further limited by pre-analytical and analytical challenges. Therefore, a weak recommendation was made to consider the clinical use of BTMs in monitoring antiresorptive or osteoanabolic pharmacotherapy in GIOP.

- *Recommendation 20: When glucocorticoids are discontinued, we recommend a re-evaluation of fracture risk to guide the decision to continue or stop GIOP prevention (strong recommendation, moderate quality evidence).*

BMD and fracture risk generally recover towards baseline levels when glucocorticoids are discontinued (9, 12, 43) although some detrimental effects may linger, particularly in post-menopausal women or patients with vertebral fractures. Some patients may qualify for continued treatment or bisphosphonate drug holidays under guidelines for post-menopausal osteoporosis (44) male osteoporosis or osteoporosis in younger persons (42) respectively. Because of their reversible effects, discontinuation of treatment with denosumab (if any) or teriparatide should always be followed by antiresorptive therapy.

## 4 Discussion

The BBC recommendations for GIOP outlined in this manuscript both update and extend our previous consensus from more than fifteen years ago (43). For example, they include Cushing syndrome and transplantation-associated osteoporosis, which are often neglected in GIOP recommendations. They are also based on an extensive literature review and a standardized grading of the strength of the recommendations and quality of the supporting evidence. We plan to update these recommendation in ten years time, or earlier if major clinically relevant changes are required.

However, we recognize several limitations of this work. In contrast to our previous guideline on post-menopausal osteoporosis (44) this work was not based on a formal umbrella systematic review (as originally planned), due to lack of time and available resources. Only PubMed was searched, therefore some other systematic reviews and meta-analyses could have been missed. Since it was based on a narrative review only, these recommendations should not be considered “guidelines”. However, we believe international guidelines already fulfill this purpose (96). Most recommendations were based on low to moderate quality evidence, highlighting the need for further research into clinical aspects of GIOP. Key areas of GIOP management with remaining uncertainty in terms of clinical evidence identified in this review are summarized in **Table 6**. We also did not include patients or stakeholders from relevant national societies (*e.g.* pulmonologists, gastroenterologists, hematologists or primary care physicians), although we plan to divulge our recommendations following publication.

**Table 6:** The research agenda: key areas in the clinical management of GIOP requiring further research.

AREA	SPECIFIC QUESTIONS
**Epidemiology**	What is the population and economic burden of GIOP and its impact on quality of life?
	What is the contribution of glucocorticoid exposure and GIOP prevention to fracture risk in conditions other than RA, IBD and asthma/COPD, *e.g.* adrenal insufficiency, dermatological conditions, polymyalgia rheumatica or giant cell arteritis, immune thrombocytopenia or autoimmune hemolytic anemias?
**Appropriate use of glucocorticoids**	What is the risk of GIOP and optimal prevention strategy with local use of glucocorticoids (*e.g.* inhaled, topical, intra-articular use)?
	What is the efficacy on disease and GIOP risk of glucocorticoid-sparing alternatives in direct comparator studies, *e.g.* with biologicals or mTOR pathway inhibitors?
	What are optimal strategies to help close the treatment gap in GIOP?
**Non-pharmacological management**	What are the optimal strategies, benefits and risks of patient education about GIOP?
	What is the risk of falls and sarcopenia associated with chronic glucocorticoid use and how can it best be assessed?
	What is the effect of lifestyle, nutritional and/or exercise interventions on fracture and falls risk in GIOP?
	Are there alternative surgical procedures which can be used safely and effectively to treat vertebral compression fractures in GIOP?
**Laboratory evaluation**	What is the value of laboratory evaluations in GIOP?
**Fracture risk assessment**	How can fracture risk assessment be improved *e.g.* using advanced imaging modalities, additional clinical risk factors, biomarkers or genetic risk profiles?
	How can fracture risk assessment and treatment decisions be improved in premenopausal women (especially those of childbearing potential) and men < 40 years of age?
**Antiresorptives and bone anabolic drugs**	What is the efficacy and safety of pharmacological GIOP prevention in endogenous hypercortisolism or diseases other than RA, IBD or transplant-associated osteoporosis?
	What is the risk of osteonecrosis of the jaw and atypical femoral fractures during GIOP treatment, and how can we best manage patients who experience these adverse events?
	What is the efficacy and safety of combination and sequential therapy as well as sclerostin inhibition in GIOP?
	What are optimal strategies for long-term treatment with bisphosphonates or denosumab or transitions between these treatments in GIOP?
**Follow-up and monitoring**	What is the clinical added value of DXA, TBS, BTMs and alternative bone strength assessment tools in the monitoring and follow-up of GIOP?

In summary, GIOP is a common secondary cause of osteoporosis in adults, resulting in considerable fracture risk, impaired quality of life and health-economic burden. A large treatment gap remains, and many glucocorticoid prescribers do not consider GIOP prevention their responsibility. Nevertheless, effective, safe and cost-effective treatment options for GIOP patients at high and very high fracture risk are available. Efforts to close the treatment gap in GIOP and implement available effective fracture prevention strategies into clinical practice in primary, secondary and tertiary care are urgently needed, and we hope these recommendations contribute to this end. In any case, these recommendations are intended to assist clinicians but not to replace individual clinical judgement.

## Author Contributions

All authors contributed to the conceptualization, methodology, data collection, writing of the original draft, editing and reviewing, and approval of the final version for publication.

## Conflict of Interest

ML has received consultancy and lecture fees from Alexion, Amgen, Daiichi Sankyo, Kyowa Kirin, Menarini, Orifarm, Sandoz, Takeda, UCB, and Will Pharma. SG has received consultancy, lecture and travel fees from Alexion, Amgen, MSD, Novartis, Takeda, UCB, and Will Pharma. CV has received travel fees from Boehringer Ingelheim. JB has received consultancy, lecture fees and travel support from Alexion, Amgen, Bayer, Sandoz, Takeda, UCB and Will-Pharma. OB has received consultancy fees from Amgen, Biophytis, IBSA, MEDA, Servier, SMB, Teva, TRB Chemedica, and UCB. EC has received consultancy fees from bioMérieux, DiaSorin, Fujirebio, IDS, Menarini, and Nittobo. SR has received travel and consultancy fees from Abbott, Bayer, Eurogenerics, Gedeon Richter, Mylan, Takeda, Theramex, UCB and Will-Pharma. EG has received travel and consultancy fees from Amgen, Alexion, Daiichi Sankyo, Sandoz, Takeda, UCB, and Will Pharma.

The remaining authors declare that the research was conducted in the absence of any commercial or financial relationships that could be construed as a potential conflict of interest.

## Publisher’s Note

All claims expressed in this article are solely those of the authors and do not necessarily represent those of their affiliated organizations, or those of the publisher, the editors and the reviewers. Any product that may be evaluated in this article, or claim that may be made by its manufacturer, is not guaranteed or endorsed by the publisher.
